# TNFAIP3 mutation causing haploinsufficiency of A20 with a hemophagocytic lymphohistiocytosis phenotype: a report of two cases

**DOI:** 10.1186/s12969-022-00735-1

**Published:** 2022-09-05

**Authors:** Nahid Aslani, Kosar Asnaashari, Nima Parvaneh, Mohammad Shahrooei, Maryam Sotoudeh-Anvari, Farhad Shahram, Vahid Ziaee

**Affiliations:** 1 Children’s Medical Center, Pediatrics Center of Excellence, Tehran, Iran; 2 Pediatric Rheumatology Society of Iran, Tehran, Iran; 3 Department of Pediatrics, Isfahan University of Medical Sciences, Isfahan, Iran; 4 Department of Pediatrics, Tehran University of Medical Sciences, Tehran, Iran; 5 Pediatric Rheumatology Research Group, Rheumatology Research Center, Tehran University of Medical Sciences, Tehran, Iran; 6 Department of Microbiology and Immunology, Laboratory of Clinical Bacteriology and Mycology, KU Leuven, Leuven, Belgium; 7 Department of Pathology, Tehran University of Medical Sciences, Tehran, Iran; 8 Behcet’s Disease Unit, Rheumatology Research Center, Shariati Hospital, Tehran University of Medical Sciences, Tehran, Iran; 9 Division of Immunology and Rheumatology, Stanford University School of Medicine, Stanford, CA USA; 10 Division of Pediatric Rheumatology, Children’s Medical Center, No. 62 Dr. Gharib St., Keshavarz Blvd, Tehran, 14194 Islamic Republic of Iran

**Keywords:** A20 haploinsufficiency, HA20, Hemophagocytic lymphohistiocytosis, HLH, Behcet’s-like disease, Autoinflammatory disorder

## Abstract

**Background:**

A20 haploinsufficiency (HA20) is a newly introduced autosomal dominant autoinflammatory disorder, also known as Behcet’s-like disease. Some of the most common symptoms of the disease are recurrent oral, genital, and/or gastrointestinal (GI) ulcers, episodic fever, musculoskeletal symptoms, cutaneous lesions, and recurrent infections. Hemophagocytic lymphohistiocytosis (HLH) is a life-threatening condition of multi-organ failure due to excessive immune activation. HLH has been reported in a few HA20 patients. Herein, we report two children with the primary presentation of HLH, with a mutation in TNFAIP3, in favor of HA20.

**Case presentations:**

Our first patient was a 4-month-old boy who presented with fever, irritability, pallor, and hepatosplenomegaly. Pancytopenia, elevated ferritin, and decreased fibrinogen levels were found in laboratory evaluation. He was diagnosed with HLH and was treated with methylprednisolone and cyclosporine. Two years later, whole exome sequencing (WES) indicated a mutation in TNFAIP3 at NM_001270507: exon3: c.C386T, p.T129M, consistent with A20 haploinsufficiency. Etanercept, a TNF inhibitor, was prescribed, but the parents were reluctant to initiate the therapy. The patient passed away with the clinical picture of cerebral hemorrhage.

The second patient was a 3-month-old boy who presented with a fever and hepatosplenomegaly. Laboratory evaluation found pancytopenia, hyperferritinemia, hypoalbuminemia, hypertriglyceridemia, and hypofibrinogenemia. With the establishment of the HLH diagnosis, he was treated with etoposide, dexamethasone, and cyclosporine, and recovered. WES results revealed a heterozygous de novo variant of TNFAIP3 (c. T824C in exon 6, 6q23.3) that leads to a proline to leucine amino acid change (p. L275P). He was treated with etanercept and has been symptom-free afterward.

**Conclusions:**

This report is a hypothesis for developing of the HLH phenotype in the presence of TNFAIP3 mutation. Our results provide a new perspective on the role of TNFAIP3 mutation in HLH phenotypes, but more extensive studies are required to confirm these preliminary results.

## Background

Hemophagocytic lymphohistiocytosis (HLH) is a life-threatening condition of multi-organ failure due to excessive immune activation. It can be presented at all ages but is most frequently reported in infants, occurring as a single or multiple recurring episodes. Fever, splenomegaly, cytopenia, hypertriglyceridemia, hypofibrinogenemia, and hemophagocytosis constitute the criteria for diagnosis of HLH [1]. HLH can be sporadic or happen in the context of a genetic disorder. In both circumstances, infection is the most common trigger [2]. Some genes are found to be associated with familial HLH [3]. Moreover, diseases that alter the immunologic function, such as immunodeficiencies and autoinflammatory syndromes, can be potential causes of HLH [4].

A20 haploinsufficiency (HA20) is a newly introduced autosomal dominant autoinflammatory disorder [5]. TNF alpha-induced protein 3 (TNFAIP3) encodes protein A20, an essential negative regulator of nuclear factor–κB (NF-κB)-mediated inflammation. A20 is an E3-ligase that also possesses deubiquitinase activity [6]. HA20, due to reduced protein levels, leads to increased activation in response to tumor necrosis factor α (TNF-α) and toll-like receptor (TLR) stimulation. Heterozygous loss-of-function mutations in A20 cause HA20 and Behcet’s-like disease in children [7].

HA20 resembles Behcet’s disease in some symptoms; however, some features in specific individuals lead to an initial diagnosis of atypical systemic lupus erythematosus with central nervous system vasculitis, anterior uveitis, colon ulcers, and autoimmune lymphoproliferative syndrome (ALPS) phenotype [8, 9]. Features of the disease include recurrent oral, genital, and/or gastrointestinal (GI) ulcers, episodic fever, musculoskeletal symptoms, cutaneous lesions, and recurrent infections. Patients with TNFAIP3 mutation can develop autoantibodies. Fluctuating levels of low-titer autoantibodies, including antinuclear antibodies, anti-double-stranded DNA (dsDNA), anti-Sm/ribonucleoprotein (RNP), anticardiolipin, and lupus anticoagulant, were found in the largest reported cohort [10–12]. Acute phase reactants tend to elevate with disease flares, but their levels are normal between flares, which is a characteristic of autoinflammatory syndromes [10].

The diagnosis of HA20 based on clinical symptoms is challenging due to various clinical manifestations in different patients. Therefore, the final diagnosis should be confirmed by genetic study and finding of TNFAIP3 mutation [10, 13, 14].

The scarcity of reported and published cases of HA20 and unknown presentations of the disease necessitate reporting the diagnosed cases of HA20 and sharing knowledge and experience in this area. Herein, we present a case of genetically confirmed HA20 with the primary presentation of HLH.

## Case presentations

### Patient one

Our patient was referred for the first time at the age of 4 months with the complaint of irritability, pallor, and intermittent fever. He had been admitted to another center for 3 weeks with lethargy, where packed cells and platelets were transfused due to the patient’s pancytopenia. Upon physical examination, the patient was conscious but irritable and appeared pale. The vital signs yielded normal results. His weight and height were appropriate for his age. Hepatosplenomegaly was found when palpating the abdomen. No other abnormality was found.

The patient was the son of two cousins, born by a normal vaginal delivery (NVD) after a normal pregnancy. He had reached developmental milestones. He had an older sister who was healthy. There was no family history of serious illnesses or early deaths.

Laboratory exams showed that the patient had pancytopenia. Hypochromia and target cells were found on the peripheral blood smear (PBS). He had normal ESR, CRP, and procalcitonin levels. Urine analysis and stool exam were normal, as well as urine, stool, and blood cultures. Rubella, cytomegalovirus (CMV), Toxoplasma, and Epstein-Barr virus (EBV) Immunoglobulin M (IgM) were negative. Considering the laboratory findings, sepsis could be ruled out. We also found a negative fluorescent antinuclear antibody (FANA) value.

Due to the findings of fever, splenomegaly, and cytopenia, HLH was a probable diagnosis. Cholesterol and triglyceride levels were checked, with results of 204 mg/dl and 687 mg/dl, respectively. AST, ALT, and ALP levels were mildly elevated. PT was normal, but PTT was elevated. Ferritin level was highly elevated, and fibrinogen level was decreased. Normal numbers of CD3+ T cell, CD4+ T cell, CD8+ T cell, CD16/56+ NK cell, and CD19+ B cell were found by flow cytometry. Nitroblue tetrazolium (NBT) test was also normal. The laboratory data are summarized in Table 1.

**Table 1:** Laboratory findings of the patient at presentation

Laboratory parameter	Result	Normal Range	Laboratory parameter	Result	Normal Range
White Blood Cells	3.4*	4-10 (*10^9^/L)	Triglyceride	687*	<150(mg/dL)
Neutrophil	2.6	2-7(*10^9^/L)	Cholesterol	204	<200(mg/dL)
Lymphocyte	0.6*	0.8-4(*10^9^/L)	PT	13	9.5-13.5(s)
Hemoglobin	9.2*	11-13.5(g/dL)	PTT	50	30-45(s)
Platelets	83000*	150-450(*10^9^/L)	INR	1	<1.2
Ferritin	3740*	30-220(ug/L)	Procalcitonin	0.2	<0.1 (ng/ml)
Fibrinogen	67*	150-350(mg/dL)	AST	62*	10-31(IU/L)
ESR	11	0-10(mm/hr)	ALT	43*	10-31(IU/L)
CRP	6	<6(mg/dL)	ALP	1328	180-1200(IU/L)

Asterisk point to the laboratory changes in favor of HLH

*ESR* Erythrocyte sedimentation rate test, *CRP* C-reactive protein, *PT* prothrombin time, *PTT* Partial thromboplastin time, *INR* International normalized ratio, *AST* Aspartate aminotransferase, *ALT* Alanine aminotransferase, *ALP* Alkaline phosphatase

The parents showed us a report of previous bone marrow aspiration, which was interpreted as probable HLH. We repeated the bone marrow aspiration, but the result was inconclusive. Hepatomegaly and splenomegaly were confirmed by sonography. Echocardiography interpretation was normal.

Since the HLH criteria were met according to HLH protocol 2004 [15] (fever, splenomegaly, cytopenia, hypertriglyceridemia, hyperferritinemia), methylprednisolone pulse therapy (30 mg/kg) was ordered for three doses in 3 days. Intravenous etoposide (5 mg/kg/dose for three doses) and oral cyclosporine (5 mg/kg/day divided into two doses) were also prescribed. After a week, the patient’s condition improved and the ferritin level decreased. The patient was discharged with a prescription of oral dexamethasone (1 mg three times a day) and cyclosporine (5 mg/kg/day divided into two doses).

When the patient was 2 years and 4 months old, the whole exome sequencing (WES) result was delivered to the parents. It indicated a mutation in TNFAIP3 at NM_001270507: exon3: c.C386T, which leads to amino acid change p.T129M, consistent with A20 haploinsufficiency. By that time, the patient was receiving etoposide every 2 weeks, oral dexamethasone (0.5 mg three times a day), and cotrimoxazole syrup (150 mg/m^2^/day three times a week), and the treatment had never been stopped since the first presentation of HLH at 4 months up to this point. Etanercept, as a TNF inhibitor, was prescribed. The family went back to their home city and did not initiate the treatment, apparently due to the high cost of the drug. We were later informed that before taking the new therapy, the patient became feverish and thrombocytopenic. He was admitted to a hospital in his home city. His condition rapidly deteriorated, and he had cerebral hemorrhage, which led to a loss of consciousness. He was comatose for 25 days and unfortunately passed away.

### Patient two

The patient was a 3-month-old boy admitted for a prolonged fever that had lasted for 2 weeks. The pregnancy was uncomplicated, and the infant was the first child of parents who were not related. On examination, the vital signs were stable. He had no syndromic features. The skin appeared normal. BCG vaccination scar and tonsils appeared normal. Hepatosplenomegaly was found. Laboratory findings before treatment are shown in Table 2. The peripheral blood smear showed a macrocytic picture, and the coagulation profile was normal, except for thrombocytopenia. Cerebrospinal fluid (CSF) analysis was normal (WBC = 0, RBC = 0, protein:20 mg/dl, glucose:60 mg/dl) and blood and CSF cultures were negative. In bone marrow aspiration, the hemophagocytosis picture was not detected. By considering the constellation of findings (i.e., fever, hepatomegaly, splenomegaly, pancytopenia, hyperferritinemia, hypoalbuminemia, hypertriglyceridemia, and hypofibrinogenemia, a provisional diagnosis of HLH was made.

**Table 2:** Laboratory findings of the second patient before treatment and four weeks after treatment

Laboratory Parameter	Before treatment	After treatment	Normal Range
White Blood Cells	960*	6510	4-10 (*10^9^/L)
Neutrophil count	240* (25.1%)	2930 (42%)	2-7(*10^9^/L)
Lymphocyte count	510* (53.1%)	2620 (40.2%)	0.8-4(*10^9^/L)
Monocyte count	140 (20.8%)	920 (14%)	120-800(*10^9^/L)
Hemoglobin	7.8*	9.7	11-13.5(g/dL)
Platelets	13,000*	240,000	150-450(*10^9^/L)
ESR	40	24	0-10(mm/hr)
CRP	101	45	<6(mg/dL)
AST	89*	51	10-31(IU/L)
ALT	52*	42	10-31(IU/L)
Triglyceride	508*	110	<150(mg/dL)
LDH	1200*	270	105-333(IU/l)
Ferritin	8490*	182	30-220(ug/L)
Fibrinogen	93*	453	150-350(mg/dL)

Asterisk points to the laboratory changes in favor of HLH

*ESR* Erythrocyte sedimentation rate test, *CRP* C-reactive protein, *AST* Aspartate aminotransferase, *ALT* Alanine aminotransferase, *LDH* Lactate dehydrogenase

Additional evaluations were performed to find the etiology of HLH in the patient. Serologic results (IgM and IgG) of CMV, toxoplasmosis, rubella, and human immunodeficiency virus (HIV) were within normal limits. Blood viral loads of EBV and CMV were undetectable, and the urine CMV load was 1975 copies/mL. Evaluation for leishmaniosis, brucellosis, tuberculosis, and mycoplasma yielded negative results.

Regarding the immunological evaluation, NBT was 100%. Normal numbers of CD3+ T cell, CD4+ T cell, CD8+ T cell, CD16/56+ NK cell, and CD19+ B cell were found by flow cytometry. The immunoglobulin levels were within normal limits. The metabolic evaluation was normal. FANA was normal. Since no underlying condition could be found for HLH, familial HLH (FHL) was confirmed, and molecular diagnosis (WES test) was indicated. It was performed on the NovaSeq 6000 platform with a reading length of 150 base pairs (bp), library type: SureSelect V6-Post, coverage of 100X, and paired-end sequencing on the extracted DNA from the peripheral blood of the patient and his parents.

The treatment with etoposide, dexamethasone, and cyclosporine A was started based on the HLH Guideline (2004), and a course of treatment with ganciclovir was also administered. About 4 weeks after the treatment, the spleen and liver returned to normal size, and laboratory findings showed nearly normal levels (Table 2).

WES results were prepared at this time. It identified a heterozygous de novo variant of TNFAIP3 (c. T824C in exon 6, 6q23.3) that leads to a proline to leucine amino acid change (p. L275P), and the CADD score was 29.1. This missense mutation has not been reported in the Human Gene Mutation Database (http://www.hgmd.cf.ac.uk/ac/all.php) and ClinVar Miner (https://clinvarminer.genetics.utah.edu). Sorting Intolerant from Tolerant (https://sift.bii.a-star.edu.sg) and PolyPhen-2 (http://genetics.bwh. harvard.edu/pph2/index.shtml) tools predicted this variant as probably deleterious. Sanger sequencing confirmed that the patient is heterozygous for this de novo mutation, while his parents are homozygous wild type.

Based on the genetic study, treatment changed to TNF-α inhibitor (etanercept) with a dose of 0.8 mg/kg per week subcutaneously, and cyclosporine A and corticosteroid were gradually discontinued. After 2 months of treatment with etanercept, leukocytosis and thrombocytosis were resolved, and erythrocyte sedimentation rate (ESR), C-reactive protein (CRP), serum ferritin, and liver enzymes gradually returned to normal values. After 6 months, he had normal growth and controlled systemic symptoms of inflammation with normal laboratory findings. At the time of writing this report, 4 years have passed since the first presentation of the disease. The patient has been completely symptom-free and is receiving etanercept every 2 weeks.

## Discussion

In this article, we have reported two cases of HA20 due to TNFAIP3 mutation that primarily manifested in early infancy with HLH. We explained the symptoms, laboratory evaluations, and treatments of these patients.

HLH is classified into familial (primary) and acquired (secondary) types [16]. Familial erythrophagocytic lymphohistiocytosis (FEL or FHL) is an autosomal recessive disease that in some patients is associated with decreased apoptosis triggering [17]. There are five subtypes of FHL: FHL type 1 is unknown in gene and its chromosomal location; FHL type 2 includes perforin gene (perforin 1 [PRF1], located at 10q21-22) mutation; FHL type 3 has the Munc 13-4 gene (unc-13 homolog D (UNC13D), located at 17q25) mutation; FHL type 4 includes mutations in STX11 (located at 6q24); and FHL type 5 has Munc 18-2 gene (syntaxin binding protein 2 [STXBP2], located at 19p13) mutation. Despite the name of FHL, the family history is often negative since the disease has recessive inheritance [18].

Many conditions can lead to the clinical manifestation of acquired HLH, including malignancies (leukemia, lymphoma, and other solid tumors), infections (viral, bacterial, or parasitic), and rheumatoid disorders [1]. Viral infections, especially Epstein-Barr virus (EBV), may trigger primary as well as secondary HLH [19]. Macrophage activation syndrome (MAS), which is defined as HLH associated with rheumatologic diseases, can be categorized as secondary HLH. Both of our patients met the European League Against Rheumatism (EULAR) Executive Committee and the American College of Rheumatology (ACR) criteria of MAS [20]. It should be noted that our first patient had anemia, yet the level of hemoglobin was 9.2 g/dl. The threshold for anemia in HLH criteria 2004 is 9 g/dl and it might be suggested that all five HLH criteria were not met in the first patient. Therefore, we suggest that the condition here be named as HLH/MAS.

High levels of IFN-γ are sufficient to induce many of the disease-associated hallmarks associated with autoinflammatory syndromes, including some of those associated with HLH. Some cytokines contribute to the pathogenesis of MAS, such as IFN-γ-producing CD8+ lymphocytes and TNF-α- and IL-6-producing macrophages [18, 21].

TNFAIP3 (OMIMI: 191163) on chromosome 6q23 encodes the A20 protein, which is a negative regulator of the TNF-nuclear factor-kB signaling pathway. Heterozygous mutation in TNFAIP3 leads to the autoinflammatory syndrome: familial Behcet’s-like disease [22]. This mutation has a vertical pattern in some families, consistent with autosomal dominant inheritance with a 50% probability of transfer from parents to their children.

In vitro experiments, in which mutant forms of the protein were overexpressed, did not lead to suppression of NF-κB activation. Cell types, which are affected by A20 deficiency in humans, include non-immune cells, such as skin fibroblasts and PBMC, monocyte-derived macrophages, and T cells, the latter of which can be induced to express excess pro-inflammatory cytokines, such as interleukin 9 (IL-9) and IL-17. Additionally, spontaneous NLR family pyrin domain containing 3 (NLRP3) inflammasome activity has been observed in cells from HA20 patients, expressing increased pro-IL-1β. As demonstrated by tumor necrosis factor receptor (TNFR) and IL-1R signaling blockade, the activity of these pathways is essential to maintain active disease [5].

Recurrent oral and genital aphthosis, GI ulcers, skin lesions, intermittent fever, ocular lesions, musculoskeletal involvement, vasculitis, neurologic symptoms, and recurrent infections are described in HA20 patients [23]. Although HA20 was first described by Zhou et al. as an autoinflammatory disorder, studies have found autoimmune-like features for the disease [22, 24]. Moreover, each patient seems to have a unique scenario. The three patients that Zhang et al. described in 2021, for instance, have their spectacular stories [25]. One patient presented with intermittent fever, diarrhea which was at times bloody, and monoarthritis which progressed into polyarthritis. The second patient manifested thoracic kyphosis, recurrent oral ulcers, and autoimmune hemolytic anemia. The third patient was referred for intermittent fever and perianal abscesses. The key to the diagnosis of many of the diagnosed cases of HA20 has been a genetic analysis in cases with obscure diagnoses. That is why reporting the cases of HA20 is mandatory for providing insights into diagnosis by summing up the presentations and laboratory evaluations.

It has been reported that several autoimmune and inflammatory disorders, including rheumatoid arthritis, systemic lupus erythematosus, psoriasis, inflammatory bowel disease, Crohn’s disease, Behcet’s disease, and type 1 diabetes, are associated with TNFAIP3 polymorphisms [5, 16, 26–28]. Our patients did not have any symptoms of Behcet’s-like or other autoimmune disorders in the first presentation and subsequent follow-ups, but their primary presentation was systemic inflammation. In addition, we did not find any positive autoantibodies, such as antinuclear antibodies, anti-dsDNA, anti-Sm/RNP, anticardiolipin, and lupus anticoagulant in our patients.

One of the unexpected presentations of HA20 is HLH. In case series of Li et al. [29], where four HA20 patients with various phenotypes are introduced, one patient is presented with macrophage activation syndrome (MAS), which is a subset of HLH. The patient had a fever, hyperferritinemia, hypertriglyceridemia, and hypofibrinogenemia. She had the c.259C > T (p.R87X) mutation in the TNFAIP3 gene (RS: NM_006290). This was the first report of HA20 patients presenting with MAS. Our patients also fulfilled the criteria for HLH. Since there are many etiologies for this catastrophic phenomenon, establishing the diagnosis at the time of presentation was impossible without genetic analysis.

Takagi et al. reported a case of a 7-month-old Japanese boy who developed a fever, skin rash, and hepatosplenomegaly with pancytopenia. The patient’s double negative T (DNT) cells were 5.1% of TCRαβ-positive cells. Immunological data and the patient’s phenotype supported the diagnosis of ALPS. In WES analysis, mutations associated with ALPS-1-like phenotypes were not identified, and de novo mutation of TNFAIP3 was detected [9]. Likewise this case, hepatosplenomegaly and pancytopenia in our patients were the primary presentations. Schulert et al. have also found a possible role for TNFAIP3 in immune dysregulation associated with HLH/MAS [30]. The patients introduced in literature with TNFAIP3 mutation and presentation of HLH/MAS are presented in Table 3.

**Table 3:** Characteristics of patients with TNFAIP3 mutation presenting with HLH/MAS

Number	Author	Gender	Age at onset	HLH features	Other features	TNFAIP3 mutation
1	Li at el	F	6.9 Y	Fever, hyperferritinemia, hypertriglyceridemia, hypofibrinognenemia, increased ALT	Oral ulcers, JIA, ILD	c.C259T, p.R87X
2	Takagi et al	M	7 M	Fever, hepatosplenomegaly, hyperferritinemia, thrombocytopenia, increased ALT	Skin rash, lymphadenopathy	c.1245_1248del (NM_001270507), p.Gln415fs
3	Our patient N. 1	M	4 M	Fever, pancytopenia, splenomegaly, hypertriglyceridemia, hyperferritinemia, hypofibrinognenemia, increased ALT	-	NM_001270507: exon3: c.C386T, p.T129M
4	Our patient N. 2	M	3 M	fever, hepatosplenomegaly, pancytopenia, hyperferritinemia, hypertriglyceridemia, hypofibrinogenemia	-	c. T824C in exon 6, p. L275P

*JIA* juvenile idiopathic arthritis, *ILD* interstitial lung disease, *ALT* alanine aminotransferase, *LDH* lactate dehydrogenase

A20 has a crucial role in downregulating NF-κB activity. Inflammatory cytokines such as tumor necrosis factor-α (TNF-α) and interleukin-1β1 (IL-1β1) impose their effects through the activity of NF-κB transcription factors. Failure to suppress NF-κB transcriptional activity results in chronic inflammation and cell death [6, 31]. Moreover, A20 is a negative regulator of NLRP3 inflammasome; deficiency of A20 triggers caspase-8-dependent enhancement of NLRP3 inflammasome activation [32–34]. Therefore, haploinsufficiency of A20, through the mechanisms of NF-κB overactivation and NLRP3 inflammasome, results in the overproduction of proinflammatory cytokines such as IL-1β, IL-6, IL-18, and TNF-α [14]. This state of cytokine storm is what we observe in the setting of HLH [35].

There is currently no standard treatment protocol for HA20. Nearly half of the patients were reported to respond well to colchicine, especially when used in combination with immunosuppressive or biological agents. Earlier initiation of biologic agents can possibly reduce the need for long-term glucocorticoid treatments [11, 14]. Therefore, the subsequent treatment plan for patients with such a specific mutation, after the initial control of inflammation, will be biological drugs, primarily anti-TNF and especially etanercept. Hematopoietic stem cell transplantation has been reported effective in patients with severe disease and cerebral vasculitis [10, 11]. Our first patient was receiving etoposide and oral corticosteroid before we got access to his genetic test, and then we prescribed etanercept as an anti-TNF drug. Unfortunately, his condition deteriorated before he had the time to begin the new therapy, and he passed away. In the case of our second patient, however, the genetic results were fortunately available just in time, and he received etanercept, which seems to have ceased the progression of the disease. We chose etanercept because it was more accessible in comparison with other biologics and since it was recommended in the literature, and continued it due to the favorable response in the patient.

The primary goal of treating secondary HLH is triggering the cause such as infection, malignancy, autoimmune disease, or autoinflammatory disorder. Moreover, it might be necessary to treat hyperinflammation apart from the etiology. Dexamethasone, etoposide, and cytokine inhibitors have been frequently used for that matter [15, 36]. The recombinant IL-1 receptor antagonist, anakinra [37], the anti-IL-6R antibody, tocilizumab [38], and the JAK inhibitor, ruxolitinib [36], are some of the biologic drugs that have been efficient in treating HLH/MAS. Considering the fact that TNFAIP3 mutation can be associated with HLH, and that HA20 can be presented with the symptoms of HLH/MAS, the biologic drugs seem to be the most reasonable choice for treatment of HA20 when HA20 is a possible trigger of HLH or is in differential diagnosis.

## Conclusions

This report is a hypothesis for developing of the HLH phenotype in the presence of TNFAIP3 mutation. Our results provide a new perspective on the role of TNFAIP3 mutation in HLH phenotypes, but more extensive studies are required to confirm these preliminary results.

## Data Availability

The datasets used and/or analyzed during the current study are available from the corresponding author on reasonable request.

## Abbreviations

HA20: Haploinsufficiency of A20
HLH: Hemophagocytic lymphohistiocytosis
WES: Whole exome sequencing
TNFAIP3: TNF alpha-induced protein 3
TNF: Tumor necrosis factor
TLR: Toll-like receptor
ALPS: Autoimmune lymphoproliferative syndrome
Ds DNA: Double-stranded DNA
NVD: Normal vaginal delivery
PBS: Peripheral blood smear
ESR: Erythrocyte sedimentation rate
CRP: C-reactive protein
LDH: Lactate dehydrogenase
AST: Aspartate aminotransferase
ALT: Alanine aminotransferase
PT: Prothrombin time
PTT: Partial thromboplastin time
CSF: Cerebrospinal fluid
NBT: Nitro blue tetrazolium test
CMV: Cytomegalovirus
EBV: Epstein-Barr virus
FEL: Familial erythrophagocytic lymphohistiocytosis
IL: Interleukin
DN: Double negative
HIV: Human immunodeficiency virus
Ig: Immunoglobulin
FANA: Fluorescent antinuclear antibody
EULAR: The European Alliance of Associations for Rheumatology
ACR: American college of rheumatology
